# Functional and Antioxidative Characteristics of Soft Wheat and Tiger Nut (*Cyperus esculentus*) Flours Binary Blends

**DOI:** 10.3390/foods13040596

**Published:** 2024-02-16

**Authors:** Svitlana Nedviha, Joanna Harasym

**Affiliations:** 1 Department of Bakery and Confectionary Technology, State Biotechnological University, Alchevskih St. 44, 61002 Kharkiv, Ukraine; nedviga_sveta@ukr.net; 2 Department of Biotechnology and Food Analysis, Wroclaw University of Economics and Business, Komandorska 118/120, 53-345 Wroclaw, Poland; 3 Adaptive Food Systems Accelerator-Science Centre, Wroclaw University of Economics and Business, Komandorska 118/120, 53-345 Wroclaw, Poland

**Keywords:** soft wheat, tiger nut, techno-functional properties, pasting, antioxidant

## Abstract

Tiger nut (*Cyperus esculentus*) or chufa is little known plant of high nutritious content. Popularized by a plant-based drink called “horchata de chufa,” it still offers a lot to research, being abundant in fat, starch, fiber and minerals and vitamins. To properly adjust this raw material to new purposes, the knowledge of crucial properties of the most basic blends like with soft wheat flour is needed. This article evaluates the techno-functional, viscometrical and bioactive characteristics of soft wheat:tiger nut blends of 5%, 10%, 15%, 20% and 25%. Granulometry, water-holding capacity (WHC), water absorption capacity (WAC), water absorption index (WAI), water solubility index (WSI), oil absorption capacity (OAC), hydrophilic/lipophilic index (HLI), color, pasting properties, total polyphenol content (TPC), antioxidant activity (DPPH), reducing sugars content and dough-rising capacity were assessed. The addition of tiger nut improved total polyphenol content of blends, however, It was observed that the addition of tiger nuts raised the total polyphenol content of the mixtures, but this was not statistically significant despite as much as 25% of tiger nuts. Oppositely, antioxidant activity was gradually improved with increasing tiger nut content. Pasting properties were impacted by tiger nut addition, lowering both pasting viscosity and trough viscosity, however, final viscosity was not particularly affected, being lowered by less than 15%. The highest water absorption was noted for 100% tiger nut both for WHC and WAC, however, WAI was the lowest for this sample. All the blends with tiger nut revealed improved dough-rising profile.

## 1. Introduction

Bakery products are some of the main sources of dietary proteins, carbohydrates, vitamins, macro and trace elements and fiber [1]. Due to its pleasant taste and important functional features, wheat flour is the major ingredient of various recipes. Enriching wheat-based food formulations with minor crops is crucial for enhancing their nutritional value and diversifying global food sources [2,3]. These minor crops can provide essential nutrients and resilience to climate change [3]. Additionally, supplementation with non-wheat flours, including those from legumes, oilseeds, and other cereals, can further improve the nutritional composition of bakery products [4,5]. Therefore, the incorporation of minor crops into wheat-based food formulations is a key strategy for addressing nutritional deficiencies and promoting biodiversity and food security.

Tiger nut (*Cyperus esculentus*), also known as chufa, is a weed plant (yellow nut sedge) of tropical and Mediterranean regions [6]. It is cultivated in north and South Africa, Spain, Portugal, Italy, South America and the United States, as well as in Ukraine [7]. The cultivation of tiger nut aims at exploitation of its sweet, almond-flavored nuts, which are also a source of edible oil. Tiger nut flour is characterized by high nutritional and biological value as it contains: 28.36 ± 0.14 g/100 g lipids, 22.36 ± 0.13 g/100 g starch, 10.4 ± 0.4 g/100 g protein, 20.2 ± 0.2 g/100 g fibers, 15.8 ± 1.3 g glucose eq./100 g sugars [8], while the amino acid profile reveals valine having the highest concentration (67.59 μg/100 g), followed by leucine (3.019 μg/100 g), phenylalanine (1.767 μg/100 g), lysine (0.946 μg/100 g), histidine (1.048 μg/100 g) and tryptophan (0.055 μg/100 g), with other amino acids in smaller amounts [9]. The composition of pressed tiger nut oil contains saturated fatty acids such as palmitic (13.5%) and stearic acid (6.3%), and most common unsaturated fatty acid is oleic acid (67.4%) [10]. Blending of tiger nut tubers into other products increases the nutritional value and gives them specific properties due to the high content of lipids, phospholipids, sterols, tocopherols (α, β and γ), dietary fibers and minerals such as K (144.80 ± 1.10 mg/100 g), Ca (94.39 ± 0.02 mg/100 g), Na (83.92 ± 0.04 mg/100 g), Fe (19.36 ± 0.54 mg/100 g), Mg (17.63 ± 0.13 mg/100 g), Cu (13.28 ± 0.05 mg/100 g) and Zn (5.18 ± 0.01 mg/100 g). Moreover, it increases the vitamin content, such as of A (53.93 ± 1.03 μg/100 g), B (27.61 ± 1.20 μg/100 g), C (31.70 ± 1.25 μg/100 g) and E (128.75 ± 0.74 μg/100 g) [11].

Tiger nut’s taste is nutty, similar to peanuts and almonds, which makes it perfect for bakery additives. Recent research has explored the potential of tiger nut as a bakery ingredient, particularly in gluten-free products. Previous investigation found that incorporating tiger nut flour in gluten-free biscuits improved their fiber and ash content, as well as their spread ratio, resulting in biscuits of superior quality [12]. Similarly, it has been demonstrated that tiger nut flour could be used in gluten-free extruded snacks, enhancing their texture and nutritional content [13]. Research also has shown that tiger nut flour can be effectively used in blends with wheat flour, with the potential to improve the nutritional and functional properties of the resulting products. A previous study found that the addition of tiger nut flour to wheat flour increased the water-holding capacity, swelling power and solubility index [14], while Chinma et al. 2010 reported an increase in protein, fat and mineral content [15]. However, the rheological properties of the dough were affected, leading to lower water absorption and mixing tolerance index. Bamigbola et al. 2016 further demonstrated that the substitution of wheat flour with tiger nut flour enhanced the nutritional quality, particularly when combined with plantain flour [16]. These studies collectively suggest that tiger nut flour can be a beneficial addition to wheat flour blends, however, they mainly focus on enhancing nutritional content.

Although studies characterizing flour with tiger nut have been conducted for more than 10 years, their scope is highly scattered and heterogeneous, focused more on testing the quality features of final products than on the complex determination of the material properties of such mixtures. There is a lack of studies systematically analyzing the techno-functional characteristics of flour mixtures and the effect of tiger nut addition on bioactive properties of such blends.

The main aim of this study was to systematically verify how the gradual addition of tiger nut flour to soft wheat flour changes its hydrophilic/lipophilic profile, viscometric and bioactive characteristics. In particular, detailed profiles of hydration properties such as water-holding capacity, water absorption capacity, water absorption index, water solubility index, swelling power and oil absorption capacity as well as the hydrophilic/lipophilic index of soft wheat:tiger nut blends with 5–25% tiger nuts were studied. Additionally, granulometry, pasting properties and dough-rising profile were studied. The complex picture is completed by the analysis of color, total polyphenol content (TPC), antioxidant activity (DPPH) and the content of reducing sugars.

To our best knowledge, this is the first study evaluating the techno-functional, viscometrical and bioactive characteristics of binary blends based on soft wheat flour containing 5–25% tiger nut. The majority of works dealing with tiger nut and wheat go straight to the chosen product without the in-depth analysis of used blends, which facilitates further prediction of the most suitable products possible to be made with them.

## 2. Materials and Methods

### 2.1. Materials and Blend Preparation

**Figure 1:**
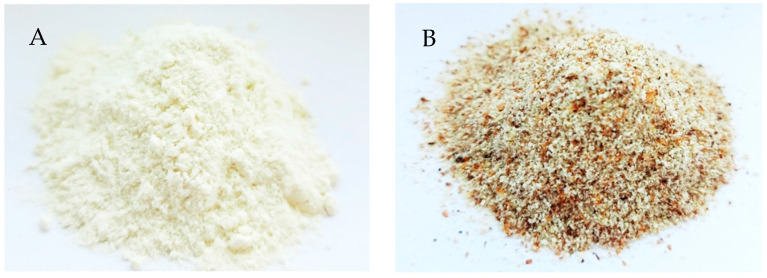
Images of flours: (**A**) wheat and (**B**) tiger nut.

Both flours were purchased from a local market: wheat flour (Novopokrovsky Bread Products Plant, Kharkiv, Ukraine) and tiger nut flour (JB NATURAL FOODS, S.L., Valencia, Spain) packaged in Ukraine. The chemical composition of tiger nut flour according to the manufacturer (per 100 g of product) was as follows: protein 7.0 g, oil 28.2 g, carbohydrates 43.0 g, fibers 11.7 g, sugars 13.3 g, vitamins: E 10.0 mg, B9 141.0 mg, minerals: K 57.0 mg, Ca 70.0 mg, Mg 870 mg, Zn 4.0 mg and Cu 0.1 mg; while for wheat it was: protein 10.3 g, oil 1.1 g, carbohydrates 70.0 g, fibers 3.5 g and sugars 0.3 g. Images of wheat flour and tiger nut flour are presented in Figure 1.

### 2.2. Flour Blend Preparation

Flour blends (in shares of 5%, 10%, 20%, 30% tiger nut flour and 95%, 90%, 80%, 70% soft wheat flour) were prepared using a rotary drum mixer (TM100, Vevor, Guangzhou, China) operating variable direction of rotation for 10 min. The 100% tiger nut flour and 100% soft wheat flour were analyzed as control samples. The moisture content of each sample, determined according to the AACC 44-19.01 method, was 13.8% (100% soft wheat), 12.6%, 12.1%, 12.4%, 12.3% (5%, 10%, 20%, 30% tiger nut flour, respectively) and 12.0% (100% tiger nut).

### 2.3. Particle Size Distribution

Particle size distribution of flours was determined according to the standard method AACC 66-20.01 with slight modifications. The samples (100 g) were sieved with an LPzE-2e vibratory sieve shaker (Multiserw Morek, Brzeźnica, Poland) at 0.65 mm vibration amplitude for 10 min with screens of 80, 106, 125, 150, 180, 200 and 250 μm. The percentage of each size of samples was calculated as the percentage vs. the initial sample weight.

### 2.4. Water Absorption Characteristics

Water-holding capacity (WHC) was determined according to the method described previously [17]. A sample of 2 g (W_s_) was weighed and suspended in 20 mL of distilled water poured previously into a pre-weighed centrifuge tube (W_t_) by spontaneous soaking and sedimentation, then was kept without any mixing for 24 h. After that, the supernatant was carefully discarded and the tubes weighed (W_ts_). WHC (expressed as g of water held per g of sample dry matter) was calculated according to Equation (1):

(1) $$WHC=\frac{W_{ts}-W_{t}}{W_{s}}\;$$

Water absorption capacity (WAC), oil absorption capacity (OAC) and hydrophilic/lipophilic index (HLI) of the samples were determined [17]. Specifically, 1 g of sample (W_S_) was mixed with 30 mL of distilled water (for WAC) or oil (for OAC) in centrifuge tubes (W_t_). After vortexing (Vortex 06-MX-S, Stargard, Poland) for 30 s, the samples were left to rest for 10 min, then the procedure was repeated twice. After centrifugation for 25 min at 3000× *g* (MPW-350, MPW, Warszawa, Poland), the supernatants were discarded, and the residual liquid was dried in an oven (Vindon Scientific, Rochdale, UK) at 50 °C for 25 min, then tubes were weighed (W_ts_). WAC (g of water/g of DM), OAC (g of oil/g of DM) and HLI were calculated using Equations (2) and (3):

(2) $$WAC;OAC=\frac{W_{ts}-W_{t}}{W_{s}}\;$$

(3) $$HLI=\frac{WAC}{OAC}$$

Determination of water absorption index (WAI), water solubility index (WSI) and swelling power (SP) proceeded according to [18]. Specifically, 1 g (W_s_) of sample was suspended in 30 mL of distilled water in centrifuge tubes (W_t_), then heated at 90 °C in a water bath (MLL147, AJL Electronics, Kraków, Poland) for 10 min. After cooling till room temperature, tubes were centrifuged at 3000× *g* for 10 min. The sediment was weighed (W_ws_) and the supernatant poured into a pre-weighed stainless steel Petri dish (W_pd_) and heated at 110 °C for 24 h in an oven (SML, Zalmed, Łomianki, Poland) to determine the solid content (W_ss_). WAI (g of water/g of DM), WSI (g of water/100 g of DM) and SP (g of water per g of DM) were calculated using Equations (4)–(6):

(4) $$WAI=\frac{W_{ws}-W_{t}}{W_{s}}$$

(5) $$WSI=\frac{W_{ss}-W_{pd}}{W_{s}\times 100}$$

(6) $$SP=\frac{W_{ws}-W_{t}}{W_{s}\;(W_{ss}-W_{pd})}$$

### 2.5. Pasting Properties

The pasting properties of samples, especially aspects of pasting viscosity like peak viscosity (PV), trough viscosity (TV), final viscosity (FV), breakdown viscosity (BD) and setback viscosity (SB), as well as pasting point time (PPT) and pasting point temperature (PPTp), were recorded via ThermoCline v. 3.17.5.15 software. The Rapid Visco Analyser (RVA 4500, Perten Instruments, Massachusetts, USA) method was used according to the AACC 76-21.01. Briefly, a temperature ramp (50 °C for 2 min, then an increase from 50 °C to 95 °C at a heating rate of 5 °C/min, maintaining at 95 °C for 5 min, cooling down at a rate of 10 °C/min back to 50 °C, holding at 50 °C for 4 min) was applied to the blend solution of 28 g in an aluminum cylinder.

### 2.6. Dough-Rising Profile

First, 50 g of dough (100 g of the blend, 1 g dry yeasts, 1.5 g of salt, and water up to 44% total water in blend) was placed in a 200 mL wide-mouth graduated cylinder, evenly spread, and a light, flat plastic foam circular cover with level top marked the initial volume. The sample was placed in a thermostat at a temperature of 35 °C. The rising profile was read every 10 min from the volume obtained by the dough, indicated by the level to which the cover rose. The breaking of the dough, meaning gas release, was assumed as the end of the cycle.

### 2.7. Color

Color was measured using a Konica Minolta CR-310 chroma meter (Ramsey, NJ, USA) connected to a data processor (DP-301) via an RS232 serial port on a personal computer [19]. To measure the color of flour, the CR-A50 accessory for granular sample measurement was used. Color parameters were taken in four replicates, while each value was a mean of four measurements. Parameters were presented as L*, a*, b*, chroma and hue.

### 2.8. Total Polyphenol Content

First, 1.0 g of each sample was mixed (1 min) with 5 mL of acidified (1% HCl) methanol/water (80:20 *v*/*v*) solvent on a vortex (MX-S, Chemland, Stargard, Poland), then agitated on a rotary shaker (MX-RD PRO, Chemland, Stargard, Poland) at room temperature for 2 h. Afterwards, extraction tubes were centrifuged at 3500× *g* for 15 min (MPW-350, MPW MED. INSTRUMENTS, Warsaw, Poland) and the supernatant was collected. The same extract was used for DPPH antioxidant activity analytics. To 20 μL of extract in a test tube, 1580 μL of distilled water was added, followed by 100 μL of Folin–Ciocalteu reagent (not diluted) and the mixture was vortexed and incubated for 6 min at room temperature (25 °C). Then, 300 μL of saturated CaCO_3_ solution was added and vortexed thoroughly until a permanent blue color was obtained. The resulting solution was incubated at 38 °C in a water bath (06-DK-98-IV, Chemland, Stargard, Poland) for 30 min in the dark. The absorbance was measured at 765 nm (SEMKO, Warsaw, Poland). Results were expressed as mg of gallic acid equivalent (GAE)/g per g of DM [17,18].

### 2.9. Antioxidant Activity (DPPH)

Previously obtained extract was used for antioxidant activity measurement using 2,2-diphenyl-1-picrylhydrazyl as a free radical source. First, 1000 μL of DPPH working solution was added to a tube, then 34.5 μL of the sample extract was added, thoroughly mixed and incubated for 20 min in the dark at room temperature. The absorbance was measured against a blank at a wavelength of 517 nm (SEMKO, Warsaw, Poland). The result was expressed as mg of Trolox equivalent (TE)/g per g of DM [17,18].

### 2.10. Reducing Sugar Content

First, 1.0 g of each sample was mixed (1 min) with 5 mL of distilled water on a vortex (MX-S, Chemland, Stargard, Poland), then agitated on a rotary shaker (MX-RD PRO, Chemland, Stargard, Poland) at room temperature for 2 h. After extraction, tubes were centrifuged at 3500× *g* for 15 min (MPW-350, MPW MED. INSTRUMENTS, Warsaw, Poland) and the supernatant was collected. The reducing sugar content of extracts was measured [20,21], taking advantage of the reducing properties of sugars towards 3,5-dinitrosalicylic acid (DNS). Then, 1 mL of DNS reagent was added to 1 mL of the sample extract and mixed thoroughly. The resulting mixture was then heated in boiling water for 5 min, then cooled to room temperature and the absorbance at 535 nm was measured (SEMKO, Warsaw, Poland). The content of monosaccharides was expressed in g of glucose equivalent per g DM.

### 2.11. Statistical Analysis

Statgraphics Centurion XVI (Statpoint Technologies, Warrenton, VA, USA) was used for the analysis of variance (ANOVA) and Fisher’s least significant difference (LSD) test was applied to evaluate significant differences (*p* < 0.05) among samples. Pearson correlation among variables was also established. All the values were presented as mean ± standard deviation. For granulometry, hydration properties, dough-rising characteristics, color, TPC and DPPH, samples were measured in triplicate, and for RVA and reducing sugars in duplicate.

## 3. Results and Discussion

### 3.1. Particle Size Distribution Characteristics

Particle size distribution of samples is shown in Table 1.

Tiger nut flour had the highest share of particles above 250 µm, which probably resulted from outer layer particle content (Figure 1) as well as higher fat content which causes agglomerating behavior. Meanwhile, soft wheat flour revealed only 5.2 ± 2.0% of particles above 250 µm. The agglomeration of particles in the flour blends caused by the stickiness of the fat was particularly evident by the increase in the proportion of the 250 µm to 200 µm fraction, as this fraction had the lowest proportion for 100% wheat and 100% tiger nut flours. However, with a rising share of tiger nut in a blend the agglomeration behavior was far more noticeable, resulting in an increase in this fraction in blends. Simultaneously, the decrease in the amount of the smallest particles in blends (starting from <180 µm) was observed and consistent which supports the agglomeration phenomenon observation. The fat-related stickiness of tiger nut during milling was also observed by other researchers [13].

### 3.2. Water and Oil Absorption Characteristics

The hydrophilic and lipophilic properties of the sample are presented in Table 2.

WHC reflects the ability of the sample to absorb water without any force other than gravity applied. WHC reveals innate imbibing capacity, showing how the sample expands while absorbing water. Although 100% soft wheat and 100% tiger nut flour were the two most absorbent samples, their blends revealed lower WHC indexes. For 100% soft wheat flour, the main ingredients that are responsible for swelling and water imbibition are starch and gluten proteins, while in tiger nut there are additional fiber particles. The different particle size distributions of the blends are shown in Table 1. The blends also presented different imbibition profiles, which are reflected in WHC values.

WAC indicates the maximum amount of water that a sample can hold when an external force is applied [17]. Among the samples, 100% tiger nut was characterized by the highest WAC value, which was again the result of imbibition capacity of the ingredients, noticed with WHC. The statistically insignificant differences observed among 100% wheat flour and all the blends suggest that the dilution of wheat content in the blends can be compensated by tiger nut addition. A previous work [22] reported similar values of WAC and OAC for the yellow variety of tiger nut. The addition of tiger nut negatively impacted WAC which was also observed in ternary blends of wheat flour:plantain flour:tiger nut flour. As WAC is negatively correlated with fat content, the addition of tiger nut, which is rich in fat, can explain the lower value.

WAI quantifies the ability of a sample to maintain water after gelatinization, WSI indicates the amount of small-molecule compounds released from a sample after gelatinization and SP shows the ability of starch to swell and maintain the volume. These three indicators characterize the sample behavior when undergoing a gelatinization process. The 100% soft wheat sample was the one with the highest WAI and SP values, while revealing the lowest WSI value. Similar values of SP for the yellow variety of tiger nut were reported by other researchers [23]. The dilution of wheat in blend samples with an increase tiger nut share resulted in a subsequent decrease in both WAI and SP. The opposite results were observed for WSI, as 100% tiger nut was the richest in small-molecule compounds being released from gel after gelatinization.

OAC reflects the lipophilic characteristics of the sample, and together with HLI it defines how the addition of certain ingredients changes the hydro-/lipophilic balance in the sample. As expected, due to the fat content, the 100% tiger nut flour sample revealed the highest OAC, however, as little as 15% tiger nut was able to statistically differentiate the blend sample from 100% soft wheat, and OAC subsequently increased with bigger shares of tiger nut. However, HLI, which represents the hydro-/lipophilic balance, of 100% samples was statistically insignificant, and high hydrophilic affinity of 100% tiger nut flour was confirmed previously by WHC and WAC indexes. The effect of wheat flour dilution and the presence of fat was most visible at 20% and 25% shares of tiger nut in blends, resulting in the lowest HLI values.

### 3.3. Pasting Characteristics

The pasting viscosity parameters of blends are presented in Table 3. The peak viscosity (PV) in all samples decreases significantly with the addition of tiger nut flour. PV is related with the ability to swell and maintain water, mainly by starch granules, so the observed behavior was in line with swelling power of samples discussed previously. The trough viscosity (TV) was the highest for the 100% soft wheat sample and the lowest for the 100% tiger nut sample. The trough viscosity identifies the breaking of starch granules as an effect of excessive imbibition. The highest value obtained for 100% soft wheat confirmed that the main viscosity creation factor in the blend slurry was wheat starch. The blends show varying TV, reflecting mainly the dilution of wheat starch in mixtures. However, a lower trough viscosity value can also be contributed by tiger nut starch and fat. A previous study showed that pasting profile viscosities of isolated tiger nut starch are very high, being higher than those for corn and potato starch [22].

Again, the final viscosity (FV) in samples was the highest for 100% soft wheat and the lowest for 100% tiger nut, showing that tiger nut is not resistant to temperature changes as setback values for the tiger nut sample were 51.0 ± 0.0 mPa*s, which, compared to the PV of 85.0 ± 0.0 mPa*s, shows substantial improvement. Other researchers also reported that final viscosity of isolated tiger nut starch reached 5094 ± 109.71 mPa*s, which can explain why the final viscosities of blends were not particularly impacted by tiger nut addition [22].

Breakdown (BD) showed a gradual decrease with rising shares of tiger nut, while setback (SB) values were improved for all the blends. That was surprising as the hindering impact of amylose chain alignment was expected during the cooling phase, however, the decrease in final viscosity was not so pronounced, being 5%, 8%, 8%, 9% and 13% for 5%, 10%, 15%, 20% and 25% tiger nut shares, respectively.

Pasting temperature was increased with tiger nut addition, resulting in a more than 20-degree difference between 100% soft wheat flour and 25% tiger nut blend. Pasting temperature is defined as a temperature at which the viscosity begins to increase during the heating ramp. Usually, a higher pasting temperature indicates that the sample, specifically starch, has a higher resistance to swelling and rupture, however, in the case of blends, it was possibly caused not only by wheat starch dilution but also by increasing fat content coming from tiger nut, which hindered water imbibition and slowed the viscosity increase. It was also confirmed by peak time which indicates the moment when PV is registered during the test. A smaller amount of starch together with a fat layer continuously hindering the water imbibition resulted in no statistically significant change among peak times of samples.

**Figure 2:**
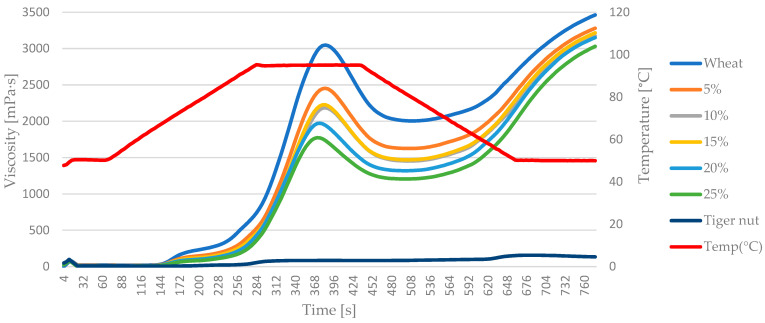
Pasting curves of wheat–tiger nut blends; 5–25%—tiger nut flour addition; lower-case letters mean values in columns are statistically different (*p* = 0.05).

Figure 2 shows a graph of the curves for mixtures of wheat flour and tiger nut flour. Pasting curves are considered a fingerprint of each sample, and the characteristic shape of the pasting curves can be associated with particular phenomena that are occurring during heating–cooling tests. Better than numerical data, pasting curves represent the similarities or differences of all the blends. Figure 2 shows how different all the wheat-containing samples are from 100% tiger nut regarding pasting profile. The increase (a slight one compared to the other blends) in viscosity after heating is also visible on the graph. The descending cascade of increased tiger nut share in blends is related to lower imbibition characteristics, while the convergence of viscosity of all blends confirms the negligible impact of tiger nut addition of firm gel forming after cooling. Similar characteristics of pasting graphs for rice and tiger nut blends were observed by other researchers [24].

### 3.4. Bioactive Characteristics

**Figure 3:**
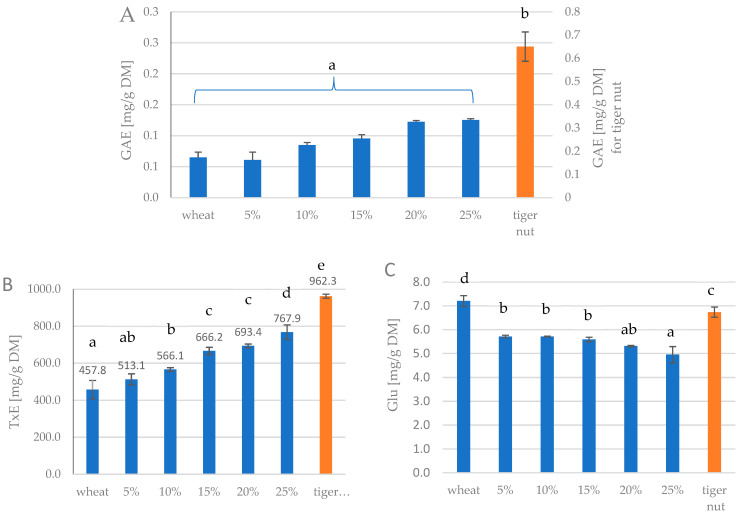
Total polyphenol content (**A**), antioxidant activity (**B**) and reducing sugar content (**C**) of wheat–tiger nut blends, 5–25%—tiger nut flour addition; lower-case letters mean values are statistically different (*p* = 0.05).

The content of polyphenolic compounds, antioxidant activity and reducing sugar content in samples are shown in Figure 3A–C.

The highest content of polyphenolic compounds is found in tiger nut flour—1.48 g, while in wheat flour—0.06 g, and in blends the TPC was gradually increased according to tiger nut shares, however still statistically non significantly. For antioxidant activity the lowest one was observed for soft wheat (468.4 mg/g DM), while tiger nut had the highest value (962.3 mg/g Dm). The addition of tiger nut positively impacted TPC and the same behavior was observed by other researchers [16], in ternary blends of wheat flour: plantain flour: tiger nut flour. Also the antioxidant activity vs. DPPH was improving as [16], which was consistent with our results. The reducing sugar content varied from 5.0 mg to 7.2 mg, with soft wheat sample being the biggest donor of reducing sugars. The addition of tiger nut gradually reduced the reducing sugar content up to 5.0 mg in sample of 25% of tiger nut share. 100% of tiger nut however, revealed 6.7 mg of reducing sugars content.

### 3.5. Color Characteristics

Color parameters of the studied wheat flours, tiger nut and mixtures are given in Table 4.

The L* value of mixtures of wheat flour and tiger nut flour ranges from 90.2 to 93.07, while the a* and b* values range from 0.6 to 0.9 and 8.97 to 9, 63. Wheat flour (control) had the highest L* values, while tiger nut flour had the lowest values. The a* values were low in the wheat flour and 5% tiger nut flour samples; tiger nut flour had the highest values. The b* value was the lowest in the 15% tiger nut flour sample and the highest in the 100% tiger nut flour sample. Using Maya seed flour caused changes in the color of bread. L* and b* values were highest in the control sample and lowest in the 30:70 Maya nut flour sample, and the a* value was lowest in the control sample and high in the 30:70 Maya nut flour sample [25].

### 3.6. Dough-Rising Characteristics

**Figure 4:**
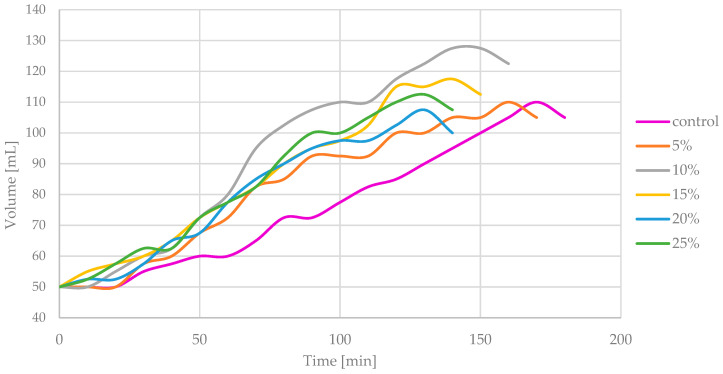
Dough-rising characteristics of control and blends; 5–25%—tiger nut flour addition.

Dough-rising characteristics are crucial factors that characterize the suitability of flour carbohydrates for yeast fermentation and ability to maintain gas bubbles [26]. An elastic and cohesive dough will rise, climbing the walls of the container up to the moment when gravitational force overcomes the internal cohesivity. Dough-rising curves of blends and wheat flour control are shown at Figure 4.

**Figure 5:**
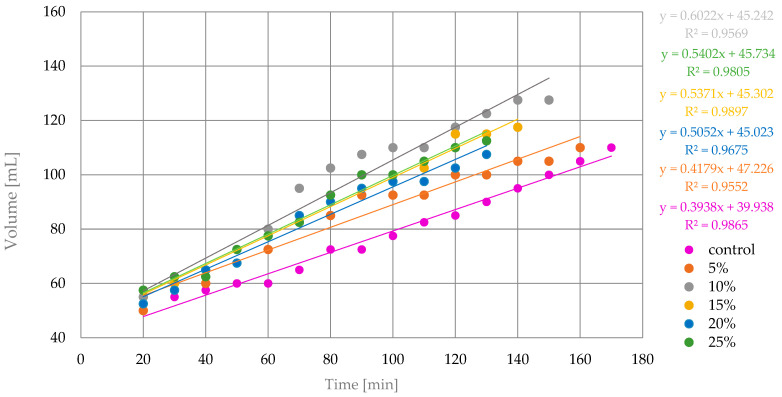
Linear adjustment of dough-rising curves of control and blends; 5–25%—tiger nut flour addition.

The control sample with 100% wheat flour had a maximum volume of 60.0 mL in 170 min. The maximum volume of 77.5 mL was found for the sample with 10% tiger nut flour; this required 150 min. The sample with 5% tiger nut flour reached a maximum volume of 60 mL in 160 min. The sample with 15% tiger nut flour reached a maximum volume of 67.5 in 140 min. For a sample with 20% tiger nut flour, it takes 130 min to reach 57.5 mL. The sample with 25% tiger nut flour reached a maximum volume of 62.5 mL in 130 min. For the linear regression approximation, the final points, representing excessive CO_2_ release and the termination of dough rising, were removed. Analysis of the characteristics of linear trend lines and equations is consistent with Figure 4. The linear adjustment is presented in Figure 5.

A substantial improvement of dough-rising power with tiger nut addition was observed. The addition of 5% tiger nut slightly improved the rising power while 10% tiger nut addition increased the power 1.5 times as the slope was 0.6022 compared to 0.3938 for control wheat flour. Additions of 15% to 25% tiger nut flour had worse performance than the 10% tiger nut binary blend but were still better than control wheat flour or even a 5% addition of tiger nut flour. The increase in rising power with tiger nut addition may be associated with two phenomena, i.e., the dilution of flour, which results in better gas-producing performance of yeast due to higher hydration of flour and increased simple sugar availability for yeasts, and higher fiber content related to bigger particles which serve as insoluble scaffolding for the rising protein–starch matrix. The domination of the first phenomenon is visible for 5% and 10% shares, which resulted in the highest slope of the linear trend, while in 15%, 20% and 25% shares the presence of fat could interfere and the wheat flour could be too diluted. However, all of the shares have more favorable dough-rising profiles, which confirms the suitability of tiger nut for structure building of leavened bakery products.

## 4. Conclusions

Tiger nut is a nutritious and underexploited raw material which is currently studied more as a healthy fat source, due to high content of unsaturated fatty acids, than a valuable structure-forming ingredient. Meanwhile, as a wheat-flour-enriching ingredient it positively impacts several techno-functional characteristics of the resulting blends.

The addition of tiger nut flour to soft wheat flour significantly impacted both nutritional and techno-functional parameters. The highest water-holding capacity and water absorption capacity were noted for 100% tiger nut flour, however, water absorption capacity was the lowest for this sample. The water solubility index rose with increasing shares of tiger nut flour and a statistically significant value was noted for the 25% share. Pasting properties were negatively impacted by tiger nut addition, lowering both pasting viscosity and trough viscosity, however, final viscosity was not particularly affected, being lowered only by shares less than 15%. Interestingly, when observing the dough-rising profile, all the blends revealed improved capacity with that of the 10% addition being the most pronounced. Total polyphenol content of blends was not significantly improved, although a rising trend was observed, which was confirmed by an increase in antioxidant activity which improved with the increase in tiger nut share.

It can be concluded that tiger nut offers a plethora of functional features which should be widely studied to popularize this highly nutritious plant. Higher shares of tiger nut improve nutritional aspects of soft wheat blends, which makes it suitable for not only bread but a variety of bakery products like cookies, muffins, cakes and doughnuts. In particular, the dough-rising-profile-enhancing capacity can improve lower-quality wheat flours, enabling their exploitation in bakery products and minimizing raw material and food loss.

## Figures and Tables

**Table 1:** Particle size profile of wheat–tiger nut blends.

Mesh Size [µm]	Wheat	5%	10%	15%	20%	25%	Tiger Nut
	[%]						
>250	5.2 ± 2.0 ^a^	47.9 ± 2.1 ^b^	47.3 ± 5.9 ^b^	48.2 ± 4.5 ^b^	51.4 ± 4.8 ^bc^	57.2 ± 2.6 ^c^	73.5 ± 0.5 ^d^
250<>200	15.3 ± 3.9 ^a^	38.1 ± 0.0 ^c^	37.6 ± 4.4 ^c^	35.3 ± 1.9 ^bc^	32.9 ± 2.4 ^bc^	29.0 ± 2.5 ^b^	20.0 ± 5.2 ^a^
200<>180	2.7 ± 0.3 ^a^	5.7 ± 0.6 ^b^	3.7 ± 0.1 ^ab^	3.9 ± 0.8 ^ab^	1.8 ± 0.0 ^a^	1.5 ± 0.1 ^a^	3.3 ± 2.7 ^ab^
180<>150	15.5 ± 3.0 ^b^	4.5 ± 1.3 ^a^	3.4 ± 0.6 ^a^	3.4 ± 0.8 ^a^	1.6 ± 0.2 ^a^	1.8 ± 0.3 ^a^	1.9 ± 1.7 ^a^
150<>125	28.8 ± 6.0 ^b^	2.0 ± 0.2 ^a^	2.1 ± 0.2 ^a^	2.6 ± 0.8 ^a^	3.1 ± 1.0 ^a^	3.9 ± 0.4 ^a^	1.0 ± 0.8 ^a^
125<>106	12.4 ± 1.2 ^d^	0.8 ± 0.3 ^ab^	2.1 ± 0.4 ^abc^	2.7 ± 2.1 ^bc^	4.3 ± 0.9 ^c^	3.8 ± 0.2 ^c^	0.2 ± 0.2 ^a^
106<>80	10.4 ± 1.6 ^d^	0.6 ± 0.3 ^ab^	2.3 ± 0.3 ^bc^	2.4 ± 1.2 ^bc^	3.6 ± 0.6 ^c^	2.4 ± 0.1 ^bc^	0.1 ± 0.2 ^a^
80<	9.7 ± 0.2 ^c^	0.4 ± 0.2 ^a^	1.5 ± 0.0 ^b^	1.5 ± 0.7 ^b^	1.3 ± 0.1 ^b^	0.5 ± 0.1 ^a^	0.1 ± 0.2 ^a^

5–25%—tiger nut flour addition; lower-case letters mean values in rows are statistically different (*p* = 0.05).

**Table 2:** The absorption characteristics of samples.

Sample	WHC	WAC	WAI	SP	WSI	OAC	HLI
	g H_2_O/g DM				g H_2_O/100 g DM	g Oil/g DM	
Wheat	2.90 ± 0.05 ^c^	1.85 ± 0.05 ^a^	6.11 ± 0.19 ^d^	6.28 ± 0.06 ^c^	885.5 ± 16.3 ^ab^	1.73 ± 0.12 ^a^	1.02 ± 0.04 ^cd^
5%	2.88 ± 0.03 ^a^	1.80 ± 0.03 ^a^	6.08 ± 0.15 ^cd^	6.32 ± 0.19 ^c^	851.8 ± 22.4 ^a^	1.80 ± 0.02 ^ab^	1.04 ± 0.06 ^d^
10%	2.80 ± 0.06 ^ab^	1.79 ± 0.02 ^a^	5.95 ± 0.23 ^cd^	6.19 ± 0.24 ^c^	860.7 ± 27.4 ^a^	1.81 ± 0.10 ^ab^	0.99 ± 0.07 ^bcd^
15%	2.84 ± 0.05 ^bc^	1.82 ± 0.03 ^a^	5.94 ± 0.14 ^cd^	6.16 ± 0.17 ^c^	874.4 ± 48.4 ^a^	1.93 ± 0.04 ^bc^	0.94 ± 0.02 ^abc^
20%	2.87 ± 0.03 ^bc^	1.80 ± 0.02 ^a^	5.76 ± 0.19 ^c^	6.01 ± 0.20 ^c^	891.4 ± 7.0 ^ab^	2.08 ± 0.11 ^c^	0.87 ± 0.04 ^a^
25%	2.88 ± 0.03 ^bc^	1.84 ± 0.09 ^a^	5.37 ± 0.09 ^b^	5.61 ± 0.09 ^b^	929.7 ± 16.5 ^b^	2.00 ± 0.10 ^c^	0.92 ± 0.06 ^ab^
Tiger nut	3.42 ± 0.02 ^d^	2.78 ± 0.21 ^b^	3.90 ± 0.30 ^a^	4.65 ± 0.38 ^a^	989.3 ± 13.8 ^c^	2.72 ± 0.08 ^d^	1.02 ± 0.06 ^cd^

5–25%—tiger nut flour addition; lower-case letters mean values in columns are statistically different (*p* = 0.05).

**Table 3:** Pasting parameters of wheat–tiger nut blends; 5%–25% tiger nut flour addition; lower-case letters mean values in columns are statistically different (*p* = 0.05).

Sample	Peak Viscosity [mPa·s]	Trough Viscosity [mPa·s]	Breakdown [mPa·s]	Final Viscosity [mPa·s]	Setback [mPa·s]	Pasting Temp [°C]	Peak Time [s]
Wheat	3047.5 ± 6.4 ^f^	2003.5 ± 16.3 ^e^	1044.0 ± 22.6 ^f^	3462.5 ± 6.4 ^f^	1459.0 ± 9.8 ^b^	69.4 ± 0.0 ^b^	6.40 ± 0.00 ^a^
5%	2458.0 ± 14.1 ^e^	1626.0 ± 67.1 ^e^	832.5 ± 53.0 ^e^	3279.5 ± 12.0 ^e^	1654.0 ± 55.2 ^c^	88.8 ± 0.1 ^c^	6.40 ± 0.10 ^a^
10%	2187.0 ± 52.3 ^d^	1483.0 ± 18.4 ^d^	704.1 ± 33.9 ^d^	3173.5 ± 43.1 ^c^	1722.0 ± 19.8 ^c^	90.1 ± 0.5 ^d^	6.40 ± 0.10 ^a^
15%	2231.5 ± 19.1 ^d^	1467.5 ± 0.7 ^d^	764.0 ± 18.4 ^cd^	3216.0 ± 5.7 ^d^	1748.5 ± 6.4 ^d^	89.7 ± 0.1 ^de^	6.37 ± 0.05 ^a^
20%	1974.0 ± 0.0 ^c^	1319.0 ± 0.0 ^c^	655.0 ± 0.0 ^c^	3151.0 ± 4.2 ^c^	1832.0 ± 4.2 ^e^	90.4 ± 0.0 ^e^	6.24 ± 0.05 ^a^
25%	1773.5 ± 13.4 ^b^	1205.5 ± 3.5 ^b^	568.0 ± 9.9 ^b^	3031.0 ± 2.8 ^b^	1825.5 ± 0.7 ^e^	91.3 ± 0.0 ^f^	6.24 ± 0.05 ^a^
Tiger nut	85.0 ± 0.0 ^a^	82.5 ± 0.7 ^a^	2.5 ± 0.7 ^a^	133.5 ± 0.7 ^a^	51.0 ± 0.0 ^a^	0.0 ± 0.0 ^a^	6.30 ± 0.14 ^a^

**Table 4:** Color parameters of wheat–tiger nut blends; 5–25%—tiger nut flour addition; lower-case letters mean values in columns are statistically different (*p* = 0.05).

Sample	L*	a*	b*
Wheat	94.77 ± 0.23 ^e^	0.60 ± 0.17 ^a^	9.57 ± 0.57 ^ab^
5%	93.07 ± 0.42 ^de^	0.60 ± 0.10 ^a^	9.27 ± 0.06 ^ab^
10%	92.00 ± 0.44 ^bcd^	0.70 ± 0.17 ^a^	9.37 ± 0.21 ^ab^
15%	92.23 ± 0.47 ^cd^	0.63 ± 0.06 ^a^	8.97 ± 0.25 ^a^
20%	90.83 ± 0.35 ^bc^	0.73 ± 0.06 ^a^	9.37 ± 0.31 ^ab^
25%	90.20 ± 0.82 ^b^	0.90 ± 0.10 ^a^	9.63 ± 0.15 ^b^
Tiger nut	73.53 ± 2.46 ^a^	3.83 ± 0.61 ^b^	17.30 ± 0.56 ^c^

## Data Availability

Data is contained within the article.
